# Gender-Related Differences in Clinical Characteristics and Outcomes of Premature Coronary Artery Disease: Insight from the FOCUS Registry

**DOI:** 10.1155/2019/6762089

**Published:** 2019-07-11

**Authors:** Ya'nan Qu, Ji'e Yang, Feng Zhang, Chenguang Li, Yuxiang Dai, Hongbo Yang, Yang Gao, Yueyi Pan, Kang Yao, Dong Huang, Hao Lu, Jianying Ma, Juying Qian, Junbo Ge

**Affiliations:** Department of Cardiology, Zhongshan Hospital, Shanghai Institute of Cardiovascular Diseases, Fudan University, China

## Abstract

**Introduction:**

Although coronary artery disease (CAD) presentations and clinical outcomes differ by sex, little is known about premature CAD (PCAD). The present analysis aimed to evaluate the gender-related differences of PCAD in an Asian population from the FOCUS registry.

**Methods:**

A total of 1397 Asian young patients with angiographically confirmed CAD undergoing drug-eluting stent implantation were included in this analysis and divided into two groups according to the genders. Patients were followed up for three years and clinical outcomes were compared between groups.

**Results:**

Young women were older and more likely to have hypertension and diabetes than men (all* p*<0.001). In contrast, males with PCAD had higher BMI and higher prevalence of current smoking as well as previous vessel revascularizations (all* p*<0.05). Men were more likely to be manifested as total occlusive lesions (*p*<0.001). Regardless of the clinical characteristics, the cumulative incidences of adverse events such as major adverse cardiovascular event (MACE), cardiovascular death, and all-cause death were not significantly different at one- or three-year follow-up (all* p*>0.05).

**Conclusion:**

Despite remarkable differences in clinical characteristics between Asian males and females with PCAD, the two groups did not differ significantly in clinical outcomes.

## 1. Introduction

Currently, the mortality of coronary artery disease (CAD) is on the decline due in part to advancement of technology in cardiovascular medicine. However, CAD remains the leading cause of mortality and morbidity worldwide [1]. The onset age of CAD gets younger and younger in both developed and developing countries [2]. Although the impact of gender on clinical outcomes after percutaneous coronary intervention (PCI) has been investigated in some studies, the issue remains complex and controversial. Importantly, relevant prior studies have been limited to small populations [3–5]. Recently, a French registry emphasized that poor in-hospital prognosis and more comorbidities were frequently associated with young females compared with males [6], while sex-related differences in mid- and long-term prognosis of premature coronary artery disease (PCAD) are less known. Therefore, the current article aims to explore sex-related differences in clinical characteristics and mid-term as well as long-term clinical outcomes of Asian patients with PCAD.

## 2. Materials and Methods

### 2.1. Study Design and Participants

The study design of the FOCUS registry has been published previously [7, 8]. Briefly, the present study is a subgroup analysis of the FOCUS registry (ClinicalTrials.gov Identifier: NCT 00868829), a large-scale, prospective, observational, and multicenter study involving 83 clinical centers in China, Thailand, and Indonesia. We followed the methods of Qu et al. (2018) [9], which described different clinical features and outcomes of Asian CAD patients in different ages. In our study, a total of 5084 individuals who were eligible for enrollment into the study received the second-generation cobalt-chromium sirolimus-eluting stent (CoCr-SES) between March 2009 and February 2010. According to the definition of PCAD in the third report of the National Cholesterol Education Program (NCEP) expert panel for the treatment of hypercholesterolemia in adults [10], a total of 1397 patients were classified as PCAD group that the onset age of CAD in males < 55 years old and in females < 65 years old. The objective of this study was to compare gender-related differences in clinical characteristics and outcomes of patients with PCAD.

All centers received the approval of medical ethics committee for the protocol. The study complied with the Declaration of Helsinki and applicable regional requirements. All patients provided written informed consents.

### 2.2. Procedures

All patients received dual antiplatelet therapy (DAPT) (usually clopidogrel and aspirin) before the operation of PCI according to the standard care of each center. Clopidogrel (75mg/day) was prescribed for at least 1 year and aspirin (100mg/day) indefinitely. Experienced senior interventional cardiologists performed the PCI procedures and visual estimation of lesion characteristics. One or more CoCr-SES was allowed to be implanted into the target vessels according to the interventional clinicians' discretions and if two or more stents were required during the procedure, CoCr-SES should be the exclusive option. Clinical follow-up was performed by either telephone communication or office visit at 30 days, 6, 12, 24, and 36 months after initial stent placement. Angiographic follow-up was not mandated in the protocol.

### 2.3. Study Endpoints and Definitions

The definition of the endpoint events of 3-year follow-up has been described in previous reports [7, 8]. The primary endpoint is major adverse cardiovascular events (MACE), including cardiac death, nonfatal myocardial infarction, and target vessel revascularization (TVR). The secondary endpoints included each individual component of the primary endpoint, all-cause death, target lesion revascularization (TLR), and definite/probable stent thrombosis (ST). Myocardial infarction (MI) was defined as typical chest pain, ST-segment or T-wave abnormalities followed by a creatinine kinase-MB level 3 times higher than the reference value. TVR was defined as repeat revascularization of the treated vessel, including any segments of the left anterior descending artery and/or left circumflex artery. All endpoints were defined according to the Academic Research Consortium. Chronic total occlusion (CTO) was defined as a complete coronary obstruction with Thrombolysis In Myocardial Infarction (TIMI) flow grade 0 with an estimated duration ≥3 months with or without visible collateral flow. Target lesion was classified as type A, B1, B2, and C according to American Heart Association/American College of Cardiology (AHA/ACC). Hyperlipidemia was defined as low-density lipoprotein cholesterol >130 mg/dl, total cholesterol >200 mg/dl, triglyceride >150 mg/dl, or high-density lipoprotein cholesterol <40 mg/dl according to the NCEP Expert Panel [10]. Definitions of other principle clinical features such as hypertension and diabetes mellitus were defined according to guidelines.

### 2.4. Data Collection and Management

Data were collected via standardized forms and subsequently entered into a computerized database. A number of procedures were set up to ensure data quality control. All centers were monitored randomly to find and correct any inaccuracies about the reported data and examine any infrared reports of the events. About 10% of the recorded data in each center were investigated by source verification based on patients' medical records and other applicable source files. All case report forms were sent to an independent clinical research organization (CCRF, Beijing, China), the data management center, where data were checked for completeness, internal consistency, and accuracy. The reports and endpoints about all events were determined by an independent clinical endpoint committee. All these measures ensured high quality of the FOCUS registry and enhanced the accuracy of the data reported in this paper.

### 2.5. Statistical Analysis

Data were presented as mean ± standard deviation (SD) for continuous variables, while all categorical variables were expressed as counts or percentage. The differences between groups were compared using Student's* t*-tests (for continuous variables) and Pearson's Chi-square test or Fisher's exact tests (for categorical variables). Cox regression survival analysis was applied to assess the associations between baseline characteristics and all-cause mortality (one year and three years) in 2 groups after adjustment to confounding risk factors. Kaplan–Meier curves were used to describe the cumulative incidence of adverse events during follow-up. All* p* values were two-tailed and values of less than 0.05 were considered to be statistically significant. Statistical analyses were conducted by using SPSS 25.0 statistical software package for windows.

## 3. Results

A total of 1397 patients with PCAD were included in this subgroup analysis and subdivided into males (872 cases) and females (525 cases) for comparison.

Table 1 shows demographic features of the study cohort. The mean (SD) age of the population surveyed was 51.3 (7.0) years. As illustrated, the most common risk factor was current smoking (62.4%) for young men and was hypertension (67%) for young women. Compared with females, males had higher BMI (*p=*0.009) and higher prevalence in prior myocardial infarction (MI) (33.3% vs. 15.8%;* p<*0.001) and previous percutaneous coronary intervention (PCI) (11.1% vs. 6.9%;* p=*0.008). In contrast, females with PCAD were more presented with diabetes mellitus (26.9% vs. 19.0%; p=0.001). Other detailed information was described in Table 1.

As shown in Table 2, a number of important differences in lesion characteristics were identified between males and females. Left anterior descending artery (LAD) was the most common intervened vessel among PCAD patients with either males or females (46.1% vs. 50.9%;* p<*0.001). Males had a higher probability of total occlusive lesion (15.8% vs. 8.7%;* p<*0.001). Lesions in males were more obviously serious in diameter stenosis (87.3±10.7% vs. 85.6±10.1%;* p*=0.001) and larger in reference diameter (3.0±0.5mm vs. 2.9±0.4mm;* p*<0.001) than those in females. However, females were more likely to have type A lesions and small vessel lesions. Other detailed information was described in Table 2.

**Figure 1:**
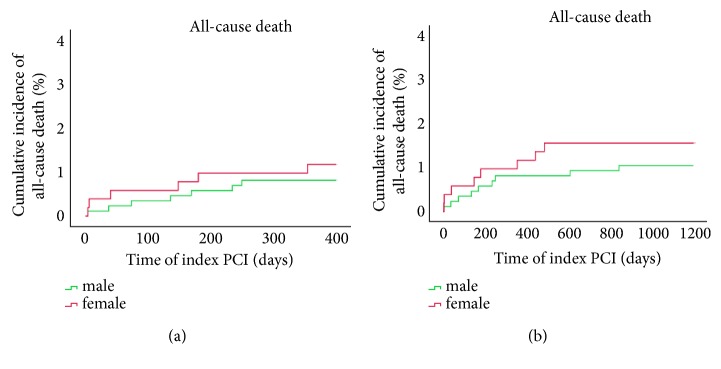
Survival analysis: Kaplan–Meier curves for all-cause death (a) at one year and three years (b) comparing males and females with PCAD.

**Figure 2:**
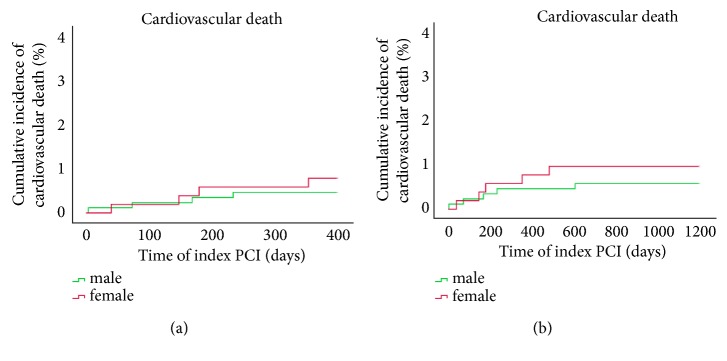
Survival analysis: Kaplan–Meier curves for cardiovascular death at one year (a) and three years (b) comparing males and females with PCAD.

**Figure 3:**
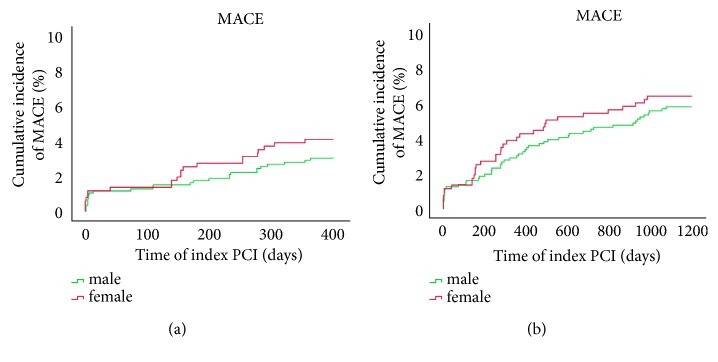
Survival analysis: Kaplan–Meier curves for MACE at one year (a) and three years (b) comparing males and females with PCAD.

Table 3 listed clinical outcomes for one-year follow-up. A total of 1385 cases were followed up (follow-up rate was 99.1%) at the end of 12 months, among which 868 cases were males and 517 cases were females. No significant differences were noted between groups with respect to the incidence of all-cause death (0.8% vs. 1.2%;* p*=0.709), cardiovascular death (0.5% vs. 0.8%;* p*=0.706), target vascular revascularization (TVR) (0.5% vs. 1.2%;* p*=0.246), and major adverse cardiovascular event (MACE) (3.0% vs. 4.1%;* p*=0.289). Yet, rates for cardiovascular death (0.5% vs. 0.8%) and MACE (3.0% vs. 4.1%), as well as other adverse events, were low. Similar trend was seen in 3-year follow-up. As shown in Table 4, complete clinical outcomes at 3 years were available in 98.9% of patients. There were no significant differences between male and female groups with respect to the outcomes of all kinds of adverse events, even after Cox regression survival analysis to adjust the confounding risk factors in Table 5. We plotted Kaplan–Meier survival curves of all-cause death, cardiovascular death, and MACE, and all remained low in both gender groups as illustrated in Figures 1–3. Though the rates were numerically increased in female group, no difference was seen either in 1-year or 3-year follow-up.

## 4. Discussion

The study of PCAD is important in the current era since the incidence of this disease is becoming higher and higher [11]. PCAD can have devastating consequences for the individual, the family, and the society [12]. Despite this serious public health, the clinical outcomes have not been adequately addressed, especially in Asian developing countries. So our study analyzes and reveals a number of gender-related differences among Asian young patients with CAD. The findings have important clinical implications.

In this cohort study, the prevalence of hypertension and diabetes in female patients with PCAD was markedly greater than in their male counterparts. These were prominent risk factors documented in other studies [13, 14], suggesting that hypertension and diabetes might play important roles in women with PCAD. High blood pressure and diabetes mellitus during pregnancy increased the risk of CAD in females [15, 16]. Similarly, the risk of CAD doubled in women with preeclampsia 5 to 10 years after pregnancy 11. These collectively contributed to the occurrence of CAD in young females. Data from the present cohort study reflected that males were more frequently smokers and overweight compared with females. Smoking was the most strongly associated risk factor of CAD and could reduce life expectancy for both sexes [17, 18]. Data from Brittany Regional Infarction Observatory (ORBI) Registry in a French study reported that young male current smokers had the highest risk for CAD and smoking particularly compromised survival in patients [6, 19]. These conclusions were also supported by the results of the INTERHEART study [20]. In agreement with another study, higher BMI was more prevalent in men [21]. It is noteworthy that there was a low proportion of family history of CAD (<10%) in our study, compared to 20% or so in the West [13]. Family history of CAD in the current study is defined as early-onset CAD of first-degree relative at <55 years of age in a male and <65 years of age in a female, respectively. The level of economic development of Asia is lower than that of the West. Moreover, the incidence of CAD has shown a rapid growth and young trend just in recent years. As a result, the incidence of early CAD in the past was low, so the proportion of family history of CAD was also lower comparing with the West.

We compared lesion characteristics in males and females who both had an early CAD in Table 2. Although left main artery disease (LAD) lesion was more likely to be associated with female population, this branch was the most common intervened vessel in both genders, as suggested by others [22]. On the contrary, the right coronary artery (RCA) lesion was more often observed in males. Meanwhile, in our study, males with PCAD in Asia were generally presented with more total occlusive lesion, mainly acute total occlusion, a strong independent predictor for repeat revascularization. Additionally, it also simultaneously indicated that the lesions in Asian younger male folks were much more unstable and tended to cause ACS, but there was no evidence to verify the difference of ACS between genders in our study.

Previous studies performed in Western countries showed that women with PCAD experienced less favorable outcomes than men [23–25]. In the present study, however, we did not find any prominent gender-related difference in the incidence of various adverse events either in 1-year or 3-year follow-up. Some reasons may account for this. Firstly, Asian men with PCAD were particularly heavy smokers as compared to the Western population, and the difference between genders in cigarette use was significantly greater than the gender gap of Western populations [26–28]. Secondly, we found that males were more vulnerable to acute total occlusive lesion in Asia which contributed to increased poor outcomes. All above risk factors led to a high incidence of adverse prognosis in Asian male patients, thus making the difference of prognoses between genders disappear. In addition, all patients in the present study were undergoing optimized PCI, and accordingly, timely and effective revascularization treatments greatly improved survival rate, resulting in low incidence of adverse events in both subsets and absence of statistical difference between groups.

The retrospective and observational nature of the study has certain limitations and strengths. Firstly, the use of CK-MB could have underestimated the detection of MI in both groups, given the superiority of troponin as a marker of myocardial injury compared to CK-MB. Secondly, some potentially confounding variables such as physical inactivity, cocaine use, unintended weight loss, and socioeconomic factors were not analyzed in the present study, but may have influenced the results. Thirdly, although a large number of baseline variables and lesion characteristics were analyzed, unmeasured confounders could influence the observed findings. However, our cohort study has strengths, including the completeness of the data set and high accuracy. It was large and contemporary and can effectively represent the distribution of Asian populations.

In conclusion, our multicentric observational registry of PCAD in Asia indicated that hypertension and diabetes mellitus were more prevalent in females, while males had a higher frequency of cigarette smoking. Although males were manifested as acute total occlusive lesions, gender was not the crucial factor for clinical outcomes of PCAD in Asian developing countries after DES implantation. It therefore seems necessary to take effective preventive measures to minimize the risk of PCAD and raise awareness of this issue to produce favorable clinical outcomes for both genders. In addition, different variables including socioeconomic factors need to be analyzed and troponin as a diagnostic marker for myocardial infarction should be used. Future investigations are warranted for a deeper insight into these notions.

## Figures and Tables

**Table 1:** Baseline characteristics: male versus female.

Variables	Total	Male	Female	*p* value
	(n=1397)	(n=872)	(n=525)	
*Demographics*				
Age (y)	51.3 ± 7.0	47.9 ± 5.2	57.0 ± 5.7	<0.001^*∗∗∗*^
BMI (kg/m2)	25.1 ± 3.0	25.3 ± 2.9	24.8 ± 3.3	0.009^*∗∗*^
*Cardiac risk factors*				
Obese	203 (14.5%)	133 (15.3%)	70 (13.3%)	0.324
Current smoking	590 (42.2%)	560 (62.4%)	30 (5.7%)	<0.001^*∗∗∗*^
Hypertension	774 (55.5%)	423 (48.5%)	351 (67.0%)	<0.001^*∗∗∗*^
Hypercholesterolemia	391 (28.0%)	250 (28.7%)	141 (26.9%)	0.465
Diabetes mellitus	307 (22.0%)	166 (19.0%)	141 (26.9%)	0.001^*∗∗∗*^
*Clinical history*				
Previous stroke	34 (2.4%)	17 (1.9%)	17 (3.2%)	0.130
Chronic renal failure	4 (0.3%)	1 (0.1%)	3 (0.6%)	0.303
LVEF≤30%	7 (0.5%)	4 (0.5%)	3 (0.6%)	1.000
Family history of CAD	104 (7.4%)	70 (8.0%)	34 (6.5%)	0.285
*Clinical indication of PCI*				
Stable CAD	198 (14.2%)	115 (13.2%)	83 (15.8%)	0.174
ACS	1199 (85.8%)	757 (86.8%)	442 (84.2%)	0.174

Values are presented as mean ± SD or n (percentage).

ACS: acute coronary syndromes; BMI: body mass index; CAD: coronary artery disease; LVEF: left ventricular ejection fractions; and PCI: percutaneous coronary intervention.

^*∗∗∗*^ *p*≤0.001, ^*∗∗*^*p*≤0.01, and ^*∗*^*p*≤0.05.

**Table 2:** Lesion characteristics: male versus female.

Variables	Total	Male	Female	*p* value
	(n=1997)	(n=1237)	(n=760)	
*Target vessel*				
LM	44 (2.2%)	33 (2.7%)	11 (1.4%)	0.071
LAD	957 (47.9%)	570 (46.1%)	387 (50.9%)	0.035^*∗*^
LCX	441 (22.1%)	271 (21.9%)	170 (22.4%)	0.810
RCA	548 (27.4%)	359 (29.0%)	189 (24.9%)	0.043^*∗*^
*Lesion class*				
Type A	342 (17.1%)	179 (14.5%)	163 (21.4%)	<0.001^*∗∗∗*^
Type B1	446 (22.3%)	283 (22.9%)	163 (21.4%)	0.456
Type B2	355 (17.8%)	235 (19.0%)	120 (15.8%)	0.069
Type C	853 (42.7%)	539 (43.6%)	314 (41.3%)	0.322
*Complex lesions*				
Total occlusive lesion	262 (13.1%)	196 (15.8%)	66 (8.7%)	<0.001^*∗∗∗*^
CTO	92 (4.6%)	62 (5.0%)	30 (3.9%)	0.270
Acute total occlusive lesion	170 (8.5%)	134 (10.8%)	36 (4.7%)	<0.001^*∗∗∗*^
Ostial lesion	177 (8.9%)	108 (8.7%)	69 (9.1%)	0.790
Bifurcation lesion	325 (16.3%)	207 (16.7%)	118 (15.5%)	0.478
Severe calcification lesion	19 (1.0%)	10 (0.8%)	9 (1.2%)	0.401
Small vessel lesion	566 (28.3%)	293 (23.7%)	273 (35.9%)	<0.001^*∗∗∗*^
Long lesion	692 (34.7%)	428 (34.6%)	264 (34.7%)	0.950
*Lesion characteristics*				
Lesion length (mm)	27.5 ± 16.4	27.7 ± 16.6	27.3 ± 16.1	0.603
Reference diameter(mm)	2.9 ± 0.4	3.0 ± 0.5	2.9 ± 0.4	<0.001^*∗∗∗*^
Lesion diameter stenosis (%)	86.6 ± 10.5	87.3 ± 10.7	85.6 ± 10.1	0.001^*∗∗∗*^
*Stent characteristics*				
Stent implanted per lesion	1.2 ± 0.5	1.2 ± 0.5	1.2 ± 0.5	0.955
Stent length (mm)	24.4 ± 6.9	24.6 ± 7.0	24.2 ± 6.9	0.007^*∗∗*^
Stent diameter (mm)	3.0 ± 0.9	3.1 ± 0.8	2.9 ± 1.1	0.319

Values are presented as mean ± SD or n (percentage). Small vessel is defined as the diameter of the vessel ≤ 2.5 mm. Long lesion is defined as the total length of the lesion ≥ 30 mm.

CTO: chronic total occlusion; LAD: left anterior descending artery; LCX: left circumflex artery; LM: left main artery; MCAD: mature coronary artery disease; PCAD: premature coronary artery disease; and RCA: right coronary artery.

^*∗∗∗*^ *p*≤0.001, ^*∗∗*^*p*≤0.01, and ^*∗*^*p*≤0.05.

**Table 3:** Clinical outcomes comparing male and female for 12 months.

Outcomes	Total	Male	Female	*p *value
(Follow-up for 1385 patients)	(n=1385)	(n=868)	(n=517)	
All cause death	13 (0.9%)	7 (0.8%)	6 (1.2%)	0.709
Cardiovascular death	8 (0.6%)	4 (0.5%)	4 (0.8%)	0.706
MI	31 (2.2%)	19 (2.2%)	12 (2.3%)	0.872
Nonfatal MI	29 (2.1%)	18 (2.1%)	11 (2.1%)	0.946
Any revascularization	27 (1.9%)	15 (1.7%)	12 (2.3%)	0.440
TVR	10 (0.7%)	4 (0.5%)	6 (1.2%)	0.246
Non-TVR	17 (1.2%)	11 (1.3%)	6 (1.2%)	0.861
MACE	47 (3.4%)	26 (3.0%)	21 (4.1%)	0.289
Definite/probable ST	7 (0.5%)	3 (0.3%)	4 (0.8%)	0.487
DAPT	1054 (76.1%)	651 (75.0%)	403 (77.9%)	0.213

Values are presented as n (percentage).

DAPT: dual antiplatelet therapy; MACE: major adverse cardiovascular event; MI: myocardial infarction; ST: stent thrombosis; and TVR: target vascular revascularization.

**Table 4:** Clinical outcomes comparing male and female for 36 months.

Outcomes	Total	Male	Female	*p* value
(Follow-up for 1382 patients)	(n=1382)	(n=865)	(n=517)	
All-cause death	17 (1.2%)	9 (1.0%)	8 (1.5%)	0.408
Cardiovascular death	10 (0.7%)	5 (0.6%)	5 (1.0%)	0.408
MI	47 (3.4%)	30 (3.5%)	17 (3.3%)	0.858
Nonfatal MI	43 (3.1%)	28 (3.2%)	15 (2.9%)	0.728
Any revascularization	75 (5.4%)	50 (5.8%)	25 (4.8%)	0.453
TVR	30 (2.2%)	17 (2.0%)	13 (2.5%)	0.498
Non-TVR	45 (3.3%)	33 (3.8%)	12 (2.3%)	0.130
MACE	83 (6.0%)	50 (5.8%)	33 (6.4%)	0.648
Definite/probable ST	9 (0.7%)	5 (0.6%)	4 (0.8%)	0.927
DAPT	147 (10.6%)	94 (10.9%)	53 (10.3%)	0.719

Values are presented as n (percentage).

DAPT: dual antiplatelet therapy; MI: myocardial infarction; MACE: major adverse cardiovascular event; ST: stent thrombosis; and TVR: target vascular revascularization.

**Table 5:** Cox regression survival analysis comparing the event rate in the 2 groups.

	12 months		36 months	
Outcomes	*χ* ^2^	*p*	*χ* ^2^	*p*
All-cause death	3.425	0.843	5.014	0.658
Cardiovascular death	4.797	0.685	4.805	0.687
MI	2.920	0.892	4.480	0.723
Nonfatal MI	2.845	0.899	5.400	0.611
Any revascularization	11.976	0.101	4.414	0.731
TVR	11.690	0.111	2.042	0.957
MACE	7.654	0.364	6.326	0.502

MACE: major adverse cardiovascular event; MI: myocardial infarction; and TVR: target vascular revascularization.

## Data Availability

The data of the FOCUS registry used to support the findings of this study have not been made available because some investigators disagreed to share.
